# Radiographic Analysis of Graft Dimensional Changes after Lateral Maxillary Sinus Augmentation with Heterologous Materials and Simultaneous Implant Placement: A Retrospective Study in 18 Patients

**DOI:** 10.3390/ma15093056

**Published:** 2022-04-22

**Authors:** Luca Comuzzi, Margherita Tumedei, Adriano Piattelli, Gianluca Tartaglia, Massimo Del Fabbro

**Affiliations:** 1 Freelance Researcher, San Vendemmiano, 31020 Conegliano Veneto, Italy; luca.comuzzi@gmail.com; 2 Department of Biomedical, Surgical and Dental Sciences, Università degli Studi di Milano, 20122 Milan, Italy; margherita.tumedei@unimi.it (M.T.); gianluca.tartaglia@unimi.it (G.T.); 3 Dental School, Saint Camillus International University for Health Sciences (Unicamillus), 00131 Rome, Italy; apiattelli51@gmail.com; 4 Dental School, University of Belgrade, 11000 Belgrade, Serbia; 5 Casa di Cura Villa Serena, Città Sant’Angelo, 65013 Pescara, Italy; 6 Fondazione Villaserena per la Ricerca, Città Sant’Angelo, 65013 Pescara, Italy; 7 IRCCS Fondazione Ca’Granda IRCCS Ospedale Maggiore Policlinico, 20122 Milan, Italy; 8 IRCCS Orthopedic Institute Galeazzi, Via Riccardo Galeazzi 4, 20161 Milan, Italy

**Keywords:** biomaterials, maxillary sinus, sinus augmentation, xenograft, lateral procedure

## Abstract

Background: This investigation aimed to radiographically assess the variations of graft dimension following maxillary sinus augmentation by the lateral approach. Methods: Eighteen patients (seven males), with a mean age at surgery of 66.5 ± 9.8 (range 52–82) years, were unilaterally treated. Thirty-five dental implants were positioned in the posterior maxilla simultaneously to grafting with heterologous biomaterials. Intraoral radiographs taken at the time of surgery, after six months, and at the longest follow-up (up to nine years after implant placement) were analyzed. The following distances were measured: mesio-distal width of the graft, vertical distance from implant apex to most coronal level of the graft, distance from the mesial aspect of the (mesial) implant to the mesial graft extension; distance from the distal aspect of the (distal) implant to the distal graft extension, and graft height along the implant axis. The dimensional changes with respect to baseline, after six months and at the longest follow-up were calculated. Results: The patient-based mean follow-up was 38.3 ± 30.1 months (range 12–108 months). The mean residual bone height at the mesial and distal aspect of the implants was 3.19 ± 2.05 mm and 2.65 ± 1.60 mm, respectively (*p* = 0.38). The mean graft width at baseline was 27.95 ± 5.23 mm, and the mean graft width reduction was 10.2 ± 12.7% (2.98 ± 3.62 mm) and 11.3 ± 14.4% (3.36 ± 4.08 mm) at six months and at the latest follow-up. The change was significant at six months (*p* = 0.005), but did not show significant further variation (*p* = 0.11). On the mesial and distal aspect, the mean graft extension decreased by 1.56 ± 2.67 mm and 0.84 ± 2.71 mm at the latest follow-up. No significant difference between mesial and distal changes was found (*p* = 0.24), suggesting that the biomaterial is resorbed homogeneously on both sides. The mean graft height was 11.92 ± 2.53 mm at baseline and decreased by 9.3 ± 9.05% (1.11 ± 1.09 mm) at six months (*p* < 0.001). Non-significant further changes were found at the latest follow-up (*p* = 0.10). Conclusions: after early remodeling, heterologous bone substitutes showed a good dimensional stability in the mid-term for maxillary sinus augmentation.

## 1. Introduction

Rehabilitation of the edentulous posterior maxilla with dental implants may often represent a clinical challenge due to the insufficient bone volume, maxillary sinus pneumatization and the low-density spongious bone [1,2]. In this clinical condition, it can be very difficult to obtain implant primary stability because of the reduced cortical bone [3]. Maxillary sinus augmentation is one of the most predictable [4] surgical procedures to reconstruct the atrophic posterior maxillary alveolar ridge and for achieving different levels of newly formed bone. The maxillary sinus floor augmentation technique can provide the necessary bone dimension for dental implants and for the achievement of osseointegration. Sinus lift can be performed through a lateral or a trans-crestal approach; in both approaches, the bone grafts are usually placed in a space obtained under the elevated maxillary sinus membrane. Different grafting materials are used for sinus floor elevation, such as autogenous bone grafts [5,6], allografts [7], alloplasts [8,9], and xenografts [10,11]. The gold standard for bone regeneration is autologous bone and it is considered the ideal scaffold due to osteogenic, osteoinductive and osteoconductive proprieties. Autologous bone grafts present some restrictions given the limited availability of the material from the donor site, and the morbidity for the patient. Recent studies have reported excellent long-term results and low postoperative morbidity using autogenous calvarial grafts [12,13]. Heterologous bone substitutes, such as bovine- or porcine-derived bone, may represent a valid alternative because of their osteoconductive properties [14]. In particular, porcine bone was found to induce the expression of osteoblast-specific genes (like osteopontin, type I collagen, and alkaline phosphatase) [15]. Furthermore, their structural characteristics seem to be similar to the human bone, they do not induce adverse reaction, and they show very low resorbability [16]. Such properties appear to be ideal for the maxillary sinus floor elevation procedure.

The dimensional modification of a maxillary sinus graft around implants has long been a subject of investigation, as graft stability may represent one of the prognostic factors for long-term implant success [17,18]. Over the years, several clinical studies investigated radiographic changes of graft height and volume in sinuses augmented using different grafting materials [18,19,20,21,22,23]. Autogenous bone grafts were found to present unpredictable resorption [24,25,26], while other popular bone substitutes like deproteinized bovine bone showed very slow or no resorption along several years [27,28]. Non-resorbable graft materials will not undergo remodeling, meaning that they will not be replaced by newly formed bone and might retain mechanical and biological properties different from the surrounding bone [29]. A certain amount of dimensional reduction of the graft is a physiological consequence of early remodeling during graft healing, and only minor changes are expected thereafter [18].

In the recent years, several heterologous materials, with different features, have been introduced in the market of bone substitutes.

The aim of the present retrospective clinical study was to evaluate the behaviour of different heterologous bone substitutes, alone or in combination, in terms of mechanical stability and bone regeneration in the sinus cavity after the maxillary sinus floor augmentation procedure via the lateral approach under conditions of daily clinical practice.

## 2. Materials and Methods

### 2.1. Study Design

The study was conducted according to the guidelines of the Declaration of Helsinki and in accordance to the Good Clinical Practice Guidelines. The Ethical Board approval was not required, given the observational retrospective nature of the study, which was conducted in a private clinic, under daily clinical practice conditions, following standard protocols. The informed consent for the retrospective study data evaluation and publishing has been obtained from all included subjects. The present retrospective study included adult patients with the need for a partial restoration in atrophic posterior maxilla, who underwent maxillary sinus augmentation procedures using a heterologous bone substitute as grafting material, and had been followed for at least 12 months after implant placement (six months after prosthesis delivery). Before the intervention, all patients signed a written consent form. Exclusion criteria were: the presence of infection in the oral cavity, such as untreated periodontal pockets on natural teeth or peri-apical fistulae; the presence of maxillary sinus infection; systemic diseases that could compromise osseointegration such as uncontrolled diabetes; radiation therapy in the craniofacial region within the previous 12 months; patients under therapy with bisphosphonates or other antiresorptive/antiangiogenic drugs; pregnancy or lactation, and smoking more than 10 cigarettes/day. Surgeries were performed by a single surgeon in a private dental practice in Conegliano Veneto (TV) Italy. Each surgery consisted of one-stage implant placement and sinus augmentation via the lateral window technique.

### 2.2. Grafting Biomaterials Used

OsteoBiol^®^ mp3^®^ (Tecnoss^®^, Giaveno, Italy) is a heterologous cortico-cancellous porcine bone mix made of 600–1000 µm or 1000–2000 µm pre-hydrated collagenated cortico-cancellous granules, properly mixed with collagen gel. The composition is 90% granulated mix, 10% collagen gel; the collagen facilitates blood clotting and the subsequent invasion of repairing and regenerative cells. The micro-porous consistency facilitates new bone tissue formation in defect sites and accelerates the regeneration process. Gradually resorbable, it preserves the original graft shape and volume (osteoconductive property) [30].

OsteoBiol^®^ Putty (Tecnoss^®^) is a cortico-cancellous heterologous porcine bone mix composed of 80% granulated mix and 20% collagen gel. OsteoBiol^®^ Putty is a paste that can be injected with a syringe between the defect walls and the implant, guaranteeing a perfect filling of the entire defect volume [31].

The OsteoBiol^®^ GTO^®^ (Tecnoss^®^) composition is 80% pre-hydrated granulated mix of porcine origin, and 20% TSV Gel. The latter is a mixture of heterologous type I and III collagen gel with polyunsaturated fatty acids and a biocompatible synthetic copolymer diluted in aqueous solution, and can be injected in the defect site. OsteoBiol^®^ GTO^®^ is gradually resorbed and it is extremely osteoconductive.

OsteoBiol^®^ Apatos^®^ (Tecnoss^®^) is a bone mixture of porcine origin, and its natural microporous consistency facilitates the formation of new bone tissue in the bone defect area, accelerating the process. OsteoBiol^®^ Apatos^®^ microcrystalline hydroxyapatite is available in cortical and mixed granules. The composition is OsteoBiol^®^ Apatos^®^ Mix: 100% heterologous cortico-cancellous mix and OsteoBiol^®^ Apatos^®^ Cortical, which is 100% cortical bone; the granulometry is 600–1000 µm and 1000–2000 µm. This material is able to guarantee adequate preservation of the grafted volume [32].

OsteoBiol^®^ Gel 40 (Tecnoss^®^) is a collagen type I and III matrix that is loaded for 60% of its volume with heterologous porcine bone granules up to 300 µm; it is rapidly and totally resorbed, its malleability and its plasticity make this product the ideal choice for sinus lift access.

Bioresorb Macro Pore (Implant Direct, Thousand Oaks, CA, USA) is an osteconductive, pure-phase beta-Tricalcium phosphate (99%) bone grafting material, available in 500–1000 microns particles size. This synthetic granular bone substitute is developed through a patented process that allows for the obtaining of a porous structure very similar to human bone. The interconnecting micro and macro porous structure promotes angiogenesis and cell transport into and throughout the granules, leading to new bone deposits within the particles.

### 2.3. Surgical Procedure

All patients underwent the same pre- and post-surgical protocol. After a primary visit and evaluation through panoramic radiographs and Cone Beam Computed Tomography Scans, all subjects treated underwent local anaesthesia performed by 4% articaine with epinephrine 1:100,000 (Pierrel, Milan, Italy). A mucoperiosteal full-thickness trapezoidal flap was elevated to expose the bone ridge and a vestibular osteotomy was performed by a bur with continuous saline irrigation. The procedure consisted of the creation of a sinus bony access window, and the Schneiderian membrane elevation was performed using appropriate surgical instruments in order to create the regenerative space. A gradually resorbable pericardium membrane, composed by heterologous mesenchymal tissue with dense collagen fibers (OsteoBiol^®^ Evolution, Tecnoss^®^) was applied covering the Schneiderian membrane in order to prevent accidental membrane perforation, and a bone graft was positioned into the neoformed cavity.

The implant drilling protocol was performed by a 2 mm diameter drill, followed by the 3 mm diameter drill under saline irrigation by a surgical motor. After implant insertion and grafting, the grafted space was covered by a resorbable pericardium membrane (OsteoBiol^®^ Evolution ) and the implant healing abutments were immediately positioned. The surgical access was sutured and an antibiotic prophylaxis was administered, consisting of amoxicillin/clavulanic acid (Augmentin, Glaxosmithkline, UK) 1 g twice daily for 6 days with pain relievers if needed. The suture removal was performed after 10 days and the control radiographs scans were conducted after three months. The prosthetic finalization was performed six months after the surgery.

### 2.4. Radiographic Evaluation

**Figure 1:**
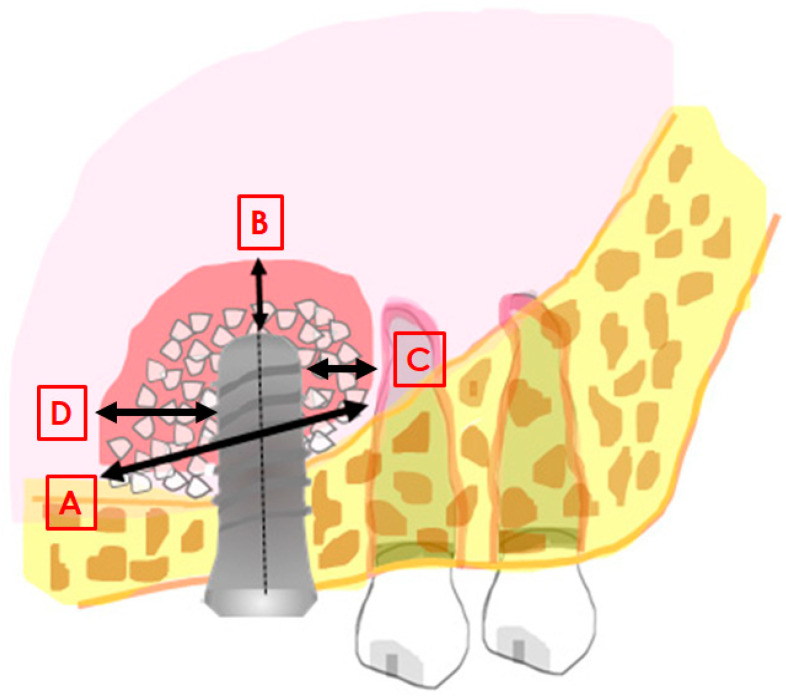
Image explaining the main distances measured in the study. (**A**): mesio-distal extension of the graft; (**B**): distance between the implant apex and the most apical level of the graft; (**C**): linear distance from the mesial aspect of the implant to the most mesial graft extension; (**D**): linear distance from the distal aspect of the implant to the most distal graft extension.

Intraoral radiographs, obtained with the parallel technique, were used for the measurements. Radiographs were analysed at the time of surgery and at the longest follow-up. The measurements were taken with the Libre Office program, version 5.1.1.3 (https://it.libreoffice.org/, accessed on 24 January 2021) by a single experienced evaluator. Prior to performing the study measurements, the evaluator underwent a calibration procedure consisting in measuring the set distances on a sample of 10 radiographs. The intra-operator error was considered acceptable when the maximum difference between consecutive measurements of the same distance was below 5%. The following distances were measured at insertion (baseline), after six months, and at the longest follow up: mesio-distal width of the graft, measured at the graft base, in contact with the original sinus floor (distance A in Figure 1); distance from implant apex to most apical level of the graft axially with the implant (B in Figure 1); linear distance from the mesial aspect of the (mesial) implant to the most mesial graft extension (C, Figure 1); linear distance from the distal aspect of the (distal) implant to the most distal graft extension (D, Figure 1). In addition, the residual ridge height was measured at the mesial and distal aspect of each implant, and the total graft height was measured axially with each implant from the most coronal aspect of the graft to the floor of the sinus referred to the native bone. The known implant length and width were used as reference for calibration of each image.

The change between baseline and the follow-up measurements was calculated both in mm and in percentage with respect to the baseline value, for each distance. All patients’ data were stored and managed using an Excel datasheet (Microsoft Office, Microsoft Corporation, Redmond, WA, USA). The personal identity of patients was anonymized.

### 2.5. Statistical Analysis

Descriptive statistics was used to describe the features of the sample, using mean values and standard deviations (SD). The difference between changes at six months and at the latest follow-up values was evaluated using a paired Student’s t-test in case of normal distributions. Otherwise, the Wilcoxon test was used. The normality of the distributions was assessed using the D’Agostino and Pearson’s omnibus normality test. Linear regression analysis between baseline values and changes, and between changes and follow-up time was undertaken, considering for each distance both the change in mm, and in percentage respect to baseline value. The software Graphpad Prism version 5.1 was used for statistical analysis. The level of significance was set at *p* = 0.05.

## 3. Results

In accordance with the inclusion criteria, 18 patients were enrolled in the present study. Seven males and eleven females, with a mean age at surgery of 66.5 ± 9.8 (range 52–82) years, received in total 35 implants in the posterior maxilla. Only four patients were smokers, and two smoked less than 10 cig/day and two smoked 10 cig/day (Table 1). Implant site, position and implant length and diameter are listed in Table 1. The type, volume, and relative proportions of each material used for maxillary sinus augmentation are reported in Table 1. For two patients (n. 4 and n. 17) the follow-up radiographs were not available or readable. Therefore, the analysis included 16 patients and 31 implants.

In Table 2 are described the main results of the dimensional evaluations. All the data are presented in mm. The mean residual bone height at the mesial and distal aspect of the implants was 3.19 ± 2.05 mm and 2.65 ± 1.60 mm, respectively (*p* = 0.38). The radiographic follow up after implant placement ranged from twelve months to nine years (mean 37.5 ± 29.8 months and 38.3 ± 30.1 months on an implant basis and patient basis, respectively).

**Figure 2:**
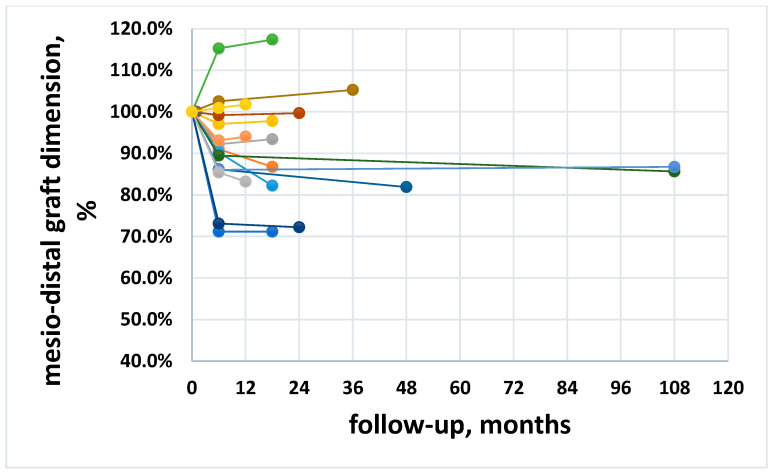
Trend of the mesio-distal dimension along follow-up for all patients, expressed in percentage. Different color lines represent different patients.

The mean graft width at baseline was 27.95 ± 5.23 mm, and the mean graft width reduction was 10.2 ± 12.7% (2.98 ± 3.62 mm) and 11.3 ± 14.4% (3.36 ± 4.08 mm) at six month follow-up and at the last follow-up, respectively. The change was significant at six months (*p* = 0.005), but there was no significant difference between six months and the latest follow-up (*p* = 0.11). Figure 2 shows the trend of mesio-distal graft dimension, expressed as percentage, for all patients. In a few cases an increase of the mesio-distal graft width was observed (Figure 2 and Table 2).

**Figure 3:**
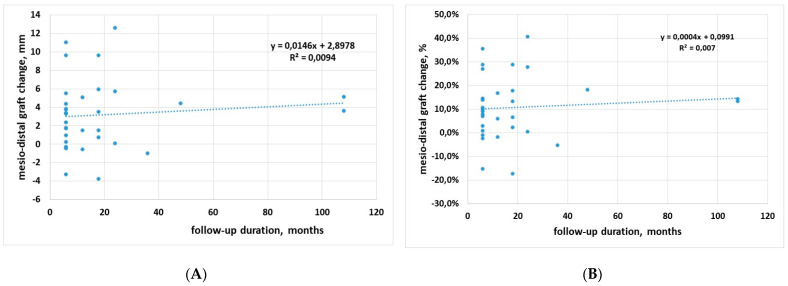
Regression analysis for mesio-distal dimension change against follow-up time. (**A**) data in mm; (**B**) data in percentage.

From 6 months onwards, there was no significant correlation between graft width change and follow-up time on a patient basis, neither with change in mm (*p* = 0.60), nor in percentage (*p* = 0.65), suggesting that after an initial reduction due to remodeling, the graft size remains unchanged over time (Figure 3A,B).

**Figure 4:**
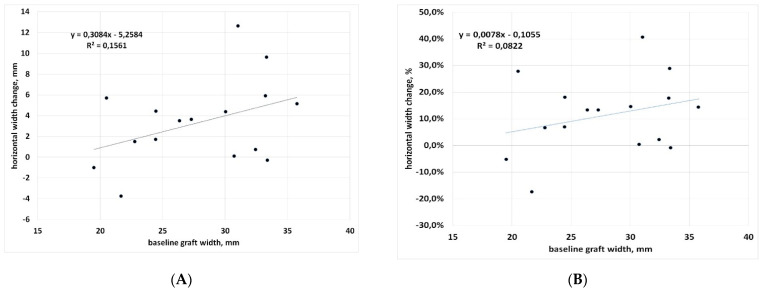
Regression analysis for mesio-distal dimension change with respect to baseline. (**A**) data in mm; (**B**) data in percentage.

Also, correlation between baseline graft width and change (in both percentage and mm) was not significant (*p* = 0.13 and *p* = 0.04, respectively), suggesting that horizontal graft changes are independent of baseline graft size (Figure 4A,B). However, a trend was observed for a greater reduction when the graft size increases (Pearson’s correlation coefficient r = 0.28 and r = 0.40 for % change and mm change, respectively), with a decrease of 0.31 mm per each mm of graft width beyond 20 mm (Figure 4A). On the mesial aspect, the mean graft extension decreased by 1.56 ± 2.67 mm, while on the distal aspect, the mean decrease was 0.84 ± 2.71 mm. In two cases (after 18 and 36 months) an increase was observed in both sides of the graft (Table 2). No significant difference between mesial and distal changes was found (*p* = 0.24), suggesting that the biomaterial is resorbed homogeneously on both sides.

The mean sinus floor augmentation (vertical height of the graft from the sinus floor) was 11.92 ± 2.53 mm at baseline and decreased by 9.3 ± 9.05% (1.11 ± 1.09 mm) at six months (*p* < 0.001). With respect to the baseline, the change at the latest follow-up averaged 10.2 ± 9.5% (1.22 ± 1.14 mm). The difference with the six month change was not significant (*p* = 0.10). Regarding the vertical extension of the graft over each implant, the mean height at baseline was 3.09 ± 1.39 mm, and at the latest follow-up, a mean reduction of 1.16 ± 1.17 mm was found (*n* = 31 implants, *p* < 0.0001).

**Figure 5:**
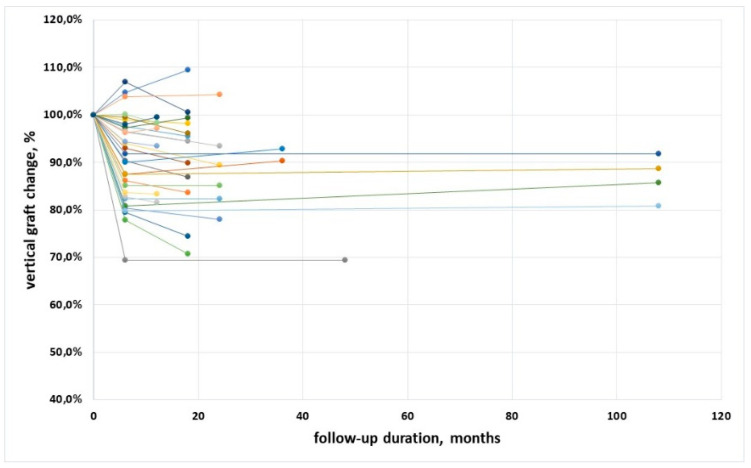
Trend of the vertical dimension along follow-up for all implants, expressed in percentage. Different color lines represent different patients.

Figure 5 shows the trend of vertical graft dimension, expressed as %, for all implants. In a few cases an increase of the mesio-distal graft height was observed (also cfr. Table 2). After an initial remodeling, no further significant changes occurred, neither on an implant basis (*p* = 0.10) nor on a patient basis (*p* = 0.64).

**Figure 6:**
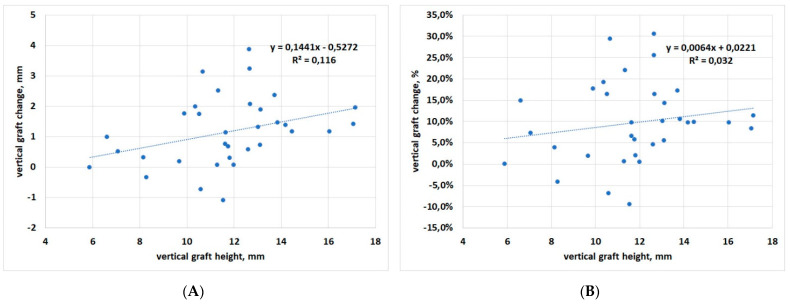
Regression analysis for vertical dimension change with respect to baseline. (**A**) data in mm; (**B**) data in percentage.

Correlation between baseline graft height and vertical change at the latest follow-up displayed slight significance when considering change in mm, but not in % (*p* = 0.04 and *p* = 0.30, respectively), suggesting that vertical graft changes are fairly independent of baseline graft height (Figure 6A,B).

## 4. Discussion

This retrospective study, conducted in a private practice setting, confirmed that the lateral approach for maxillary sinus augmentation with different grafting materials of various origin (xenografts of porcine origin and alloplasts, used alone, in combination among them or (only in one case) with autogenous bone) is a predictable procedure. In this study, the same surgeon performed all operations, and the same assessor made all measurements on the radiographs, ensuring minimization of the results’ variability. An interesting finding of the present study is that graft resorption in both the mesio-distal and the cranio-caudal dimension seems to be unaffected by the follow-up duration. In fact, the slope of the regression in Figure 2 and Figure 4 was not significantly different from zero. This would suggest that, once graft remodeling has occurred during graft healing in the first six months, the graft dimensions tend to become stable, with minimal changes over time. The mean change in the mesio-distal and in the cranio-caudal direction was about 11.3% and 9.7% of the respective graft dimensions at placement. The mean graft height apical to the implant apex averaged 3.09 mm at insertion and 1.93 mm after a mean follow-up of 37.5 months, warranting apical protection in the large majority of cases. In three out of 35 cases, the graft leveled at the implant apex, and only in one case was the level of the graft positioned 1 mm below the apex of the implant, although it did not compromise implant function. This is not surprising, as successful sinus floor elevation using the lateral window approach was reported even in the absence of graft positioning [33]. The long-term success of the graftless procedure, however, is yet to be determined, while it has been well demonstrated for the classical lateral window approach by using a multitude of grafting materials [34]. In our opinion, even if the graftless protocol may allow implant osseointegration with the formation of new bone in the space below the lifted membrane, implants completely surrounded by a grafting material are better isolated and protected, and may function undisturbed for a long time.

In the present study, no comparison in graft dimensions’ change among the different materials has been evaluated, because several materials and combinations were used, and the sample size for each of them was low, as can be seen in Table 1, thereby preventing a reliable comparison. The heterogeneity of the biomaterials used and their associations can be regarded as a major limitation of the present study. We noted that, in the cases showing an increase of the apical thickness of the graft (vertical growth), either OsteoBiol^®^ GTO^®^ or OsteoBiol^®^ Apatos^®^ were present in the graft. However, the same materials were also used in cases showing the largest vertical resorption, suggesting that factors other than the grafting material may contribute to dimensional changes of the graft. This finding is certainly interesting but needs to be confirmed by specific investigations with a higher number of cases. The largest mesio-distal resorption (more than 12 mm) was observed in a case using OsteoBiol^®^ Putty alone, but in another case the same material was associated with minimal resorption (about 1 mm). Of course, two cases are not sufficient to draw indications on the performance of this material. Another interesting finding is that in both the cranio-caudal and the mesio-distal dimension, there was a trend for larger changes to be associated with larger grafts: the linear regression slope of Figure 4 and Figure 6 was positive in both cases, but significant only for the vertical dimension, considering the changes in mm (Figure 6A). Indeed, greater vertical resorption with higher grafts is not an unexpected finding, as also reported in a recent study on transcrestal sinus floor elevation [35]. Recent studies on maxillary sinus graft regeneration, based on histomorphometric analysis, reported that new bone formation follows a gradient from the native bone (the pristine sinus floor) towards the most cranial part of the augmentation region [36,37]. They observed that when increasing the distance from the sinus floor, which represents the main source of precursor cells, nutrients and oxygen for the graft, the amount and quality of newly formed bone in the graft decreases. Therefore, the risk of resorption will be higher in the most apical parts of the graft, and will increase in the highest grafts. A recently published systematic review of randomized trials on maxillary sinus augmentation evaluated the effect of the height of residual bone and the size of the graft in the vertical dimension on graft shrinkage along time, and found results similar to the present study [38]. In particular, this systematic review suggested that minimizing the augmentation volume might have favourable effects on graft healing and stability, especially when xenografts and alloplastic grafts are used. Regarding the observed trend for greater remodeling in wider grafts in the mesio-distal dimension along the pristine sinus floor on both sides of the implant, the wider the graft, the greater the detachment of the membrane and the perturbation of the sinus physiology. We may hypothesize that, as a reaction to the stress caused to the sinus membrane by the elevation procedure, and the sinus space reduction following graft placement, an adaptation of the graft volume will follow, with resorption of the peripheral regions in both the vertical and horizontal dimension, allowing for sinus space expansion, until a new sinus volume is reached. Of course, such a hypothesis needs to be verified by specific studies with a larger sample size.

One major limitation of the present study is that all measurements were based on bi-dimensional intraoral radiographs. We acknowledge that 3D reconstructions can allow for a more precise representation of the graft extension and the remodeling pattern as well. Nevertheless, multiple assessments of the patients with computed tomography increase the x-ray exposure and cannot be justified in daily clinical practice. Studies using multiple CT assessment must require ethical approval. Furthermore, in the pool of patients considered for the present study, some of which have been treated more than 10 years ago, only periapical radiographs were available at follow-up.

Another limitation of the present retrospective study was represented by the low number of samples for each of the different biomaterials used, which prevents a sound comparison. In order to compare the performance of different materials, specific studies with wider sample size and long-term follow-up are needed. In future studies, it would be desirable to introduce stem cells in the clinical practice due to their beneficial role in bone regeneration procedures [39].

In conclusion, the data of this study performed under daily practice conditions suggest that in maxillary sinus augmentation with a lateral approach, xenogeneic bone substitutes of porcine origin, combined or not with alloplastic graft materials, undergo a resorption pattern consisting of an early remodeling, followed by a phase of stability. The amount of change appears to be related to the baseline dimension of the graft. The remodeling pattern of the graft was compatible with durable implant support and protection.

## Figures and Tables

**Table 1:** Main features of the patients, grafts and implants.

Pat ID	Smoker (Y/N) N. cig/day	Age, Years	Gender	Graft Material(s) (Volume %)	Implant Site	Implant Length, mm	Implant Diameter, mm
#1	N	53	M	OsteoBiol^®^ GTO^®^, Bioresorb 0.8 cc (80:20)	16	10	4.3
					15	10	4.3
#2	N	71	M	OsteoBiol^®^ Apatos^®^, Bioresorb 0.8 cc (90:10)	17	11	4.1
					18	11	4.1
#3	10	65	F	OsteoBiol^®^ GTO^®^, Bioresorb 1 cc (80:20)	16	9	5
#4	10	51	F	OsteoBiol^®^ GTO^®^, OsteoBiol^®^ Apatos^®^, OsteoBiol^®^ mp3^®^ 1.5 cc (40:40:20)	16	11	4.1
					17	11	4.1
#5	8	71	M	OsteoBiol^®^ GTO^®^, Bioresorb 0.8 cc (80:20)	24	11	4.1
					25	11	4.1
					26	11	4.1
#6	N	52	F	OsteoBiol^®^ GTO^®^ Bioresorb 1.8 cc (80:20)	24	11	3.8
					25	11	3.8
					26	11	4.5
#7	N	63	F	OsteoBiol^®^ GTO^®^ 0.5 cc	16	11	4.1
#8	N	72	M	OsteoBiol^®^ Apatos^®^ 0.5 cc, OsteoBiol^®^ Gel 40 0.3 cc	26	11	4.5
#9	N	82	M	OsteoBiol^®^ Apatos^®^, Bioresorb (80:20) 1 cc	25	11	3.8
					26	11	4.5
					27	11	4.5
#10	N	57	M	OsteoBiol^®^ Putty 0.6 cc	26	11	4.5
					27	11	4.5
#11	N	69	F	OsteoBiol^®^ Putty 0.8 cc	25	11	3.8
					26	11	4.5
#12	N	80	F	OsteoBiol^®^ Apatos^®^ 0.5 cc, OsteoBiol^®^ mp3^®^ 0.5 cc	16	11	4.5
#13	N	72	M	OsteoBiol^®^ mp3^®^ 0.7 cc	26	11	4.5
					27	11	4.5
#14	5	70	F	OsteoBiol^®^ mp3^®^ 0.8 cc, Bioresorb 0.2 cc	26	11	4
					27	11	5
#15	N	52	F	OsteoBiol^®^ GTO^®^ 0.8 cc, Bioresorb 0.2 cc (80:20)	26	11	4.1
					27	11	4.1
#16	N	79	F	OsteoBiol^®^ GTO^®^ 1 cc	16	12	4.1
					17	11	4.1
#17	N	69	F	OsteoBiol^®^ Apatos^®^ 0.5cc, OsteoBiol^®^ GTO^®^ 0.4cc, autologous bone 0.1 cc	16	11	3.8
					17	12	4.1
#18	N	70	F	OsteoBiol^®^ Apatos^®^ 0,5 cc, OsteoBiol^®^ GTO^®^ 0.5 cc	16	12	4.1
					17	12	4.1

**Table 2:** Results of the dimensional evaluations for 16 patients. Data are expressed in mm.

Pat. No.	RX Latest Follow-Up	Implant Site	Overall Graft Width Baseline, mm	Graft Width Latest Change, mm *	Mesial Graft Extension, mm	Mesial, Latest Change, mm	Distal Graft Extension, mm	Distal, Latest Change, mm *	Vertical Distance Implant Graft at Baseline, mm	Vertical, Latest Change, mm *
1	18 m	16	33.33	9.61	-	-	9.67	3.68	3.3	0.57
		15			8.78	2.69	-	-	3.06	1.73
2	18 m	17	26.37	3.49	5.77	0.58	-	-	4.47	0.72
		18			-	-	6.27	0.59	1.86	0.18
3	18 m	16	22.78	1.5	8.85	0.37	8.32	2.01	2.46	−1.09
5	18 m	24	32.45	0.72	6.11	1.27	-	-	4.37	3.13
		25			-	-	-	-	6	3.23
		26			-	-	1.56	−5.72	2.11	1.31
6	18 m	24	33.25	5.91	4.27	2.74	-	-	3.04	1.38
		25			-	-	-	-	1.64	0.06
		26			-	-	6.64	1.14	1.48	−0.73
7	18 m	16	21.69	−3.77	9.27	−2.19	7.59	−2.23	3.51	0.07
8	24 m	26	20.52	5.7	7.25	3.44	9.24	3.55	1.75	1.75
9	24 m	25	30.75	0.1	4.03	−0.55	-	-	1.28	−0.35
		26			-	-	-	-	2.9	0.76
		27			-	-	7.17	0.57	3.63	1.46
10	24 m	26	31.04	12.62	12.4	8.06	-	-	1.33	2.5
		27			-	-	3.91	2.36	0.98	0.98
11	36 m	25	19.51	−1.03	1.64	−0.78	-	-	1.91	0.51
		26			-	-	6.7	−0.44	2.36	1.13
12	48 m	16	24.47	4.43	10.15	0.07	10.31	4.62	3.87	3.87
13	108 m	26	35.77	5.13	12.3	4.2	-	-	5.64	1.95
		27			-	-	10.41	−0.88	5.2	1.41
14	108 m	26	27.31	3.62	6.47	1.39	-	-	4.21	1.88
		27			-	-	4.27	1.97	4.4	1.99
15	12 m	26	24.46	1.69	4.32	−1.67	-	-	1.23	0.31
		27			-	-	8.87	4.23	3.73	2.36
16	12 m	16	30.05	4.38	9.64	4.94	-	-	4.71	2.07
		17			-	-	8.27	0.97	2.17	0.67
18	12 m	16	33.41	−0.31	10.42	0.47	-	-	4.27	−0.01
		17			-	-	10.25	−1.03	3.06	0.29

* Positive values for change indicate reduction, negative values indicate increase; m = months.

## Data Availability

The authors are available to share the data upon request.
